# Modulation of Physiological Stress Response of *Triticum aestivum* L. to Glyphosate by Brassinosteroid Application

**DOI:** 10.3390/life11111156

**Published:** 2021-10-29

**Authors:** Elena Shopova, Zornitsa Katerova, Liliana Brankova, Ljudmila Dimitrova, Iskren Sergiev, Dessislava Todorova, Neveen B. Talaat

**Affiliations:** 1 Institute of Plant Physiology and Genetics, Bulgarian Academy of Sciences, 1113 Sofia, Bulgaria; kostei@abv.bg (E.S.); lbrankova@abv.bg (L.B.); dim.lyudmila@gmail.com (L.D.); iskren@bio21.bas.bg (I.S.); dessita@bio21.bas.bg (D.T.); 2 Department of Plant Physiology, Faculty of Agriculture, Cairo University, Giza 12613, Egypt; neveenbt@yahoo.com

**Keywords:** wheat, herbicide, brassinosteroids, abiotic stress

## Abstract

The potential of brassinosteroids to modulate the physiological responses of winter wheat (*Triticum aestivum* L.) to herbicide stress was evaluated. Young winter wheat seedlings were treated with 24-epibrassinolide (EBL) and 24 h later were sprayed with glyphosate. The physiological responses of treated plants were assessed 14 days after herbicide application. Wheat growth was noticeably inhibited by glyphosate. The herbicide application significantly increased the content of the stress markers proline and malondialdehyde (MDA) evidencing oxidative damage. The content of phenolic compounds was decreased in the herbicide-treated plants. Slight activation of superoxide dismutase (SOD) and catalase (CAT) and considerable increase of glutathione reductase (GR) and guaiacol peroxidase (POX) activities were found. Increased POX and glutathione S-transferase (GST) activities were anticipated to be involved in herbicide detoxification. Conjugation with glutathione in herbicide-treated plants could explain the reduction of thiols suggesting unbalanced redox state. EBL application did not alter the plant growth but a moderate activation of antioxidant defense (POX, GR, and CAT activities and phenolic levels) and detoxifying enzyme GST was observed. The hormonal priming provoked a slight decrease in MDA and proline levels. The results demonstrate that EBL-pretreatment partly restored shoot growth and has a potential to mitigate the oxidative damages in glyphosate-treated plants through activation of the enzymatic antioxidant defense and increase of the phenolic compounds.

## 1. Introduction

Herbicides are often used in modern agricultural practice to manage weed growth. Glyphosate (N-(phosphonomethyl)glycine) use has increased 15-fold from 1994 to 2014 on a worldwide scale [1]. It is the most extensively used herbicide, consequently, weed resistance to its usage has emerged. As a result, non-target contamination occurred bringing negative outcomes for a number of native plant species [2,3,4,5,6,7,8]. The introduction of glyphosate-resistant crops in agricultural practice caused an additional rise in the use of the herbicide in soybean, corn, cotton, and wheat cultivation [3,9]. Glyphosate is a broad-spectrum, non-selective herbicide, killing all affected plants. It inhibits the plastidic 5-enolpyruvylshikimate-3 phosphate synthase, a key enzyme in the shikimate pathway. Consequently the biosynthesis of aromatic amino acids and their essential derivatives (hormones, enzyme cofactors, etc.) is blocked [2,3,10]. The glyphosate principal metabolite, aminomethylphosphonic acid, which is admitted to be a phytotoxin, may occur in some plants and enhance the negative effects of the herbicide [10,11]. Due to the cation chelator features of glyphosate, it may block important enzymatic cofactors and affect enzymatic antioxidants and photosynthesis [10,12]. The boost of reactive oxygen species (ROS) in glyphosate-treated plants leading to oxidative stress is believed to be due to the inhibition of the shikimate pathway [5,10,13,14,15,16,17]. The plants treated with this herbicide exhibited stunted growth and impaired photosynthesis [5,10,17].

Oxidative stress is a common reaction response to all types of stressors, including xenobiotics and glyphosate in particular [13,18,19]. In brief, the equilibrium between ROS generation and induction of different defense systems on the molecular, metabolic (including proteomic) and physiological levels (including non-enzymatic and enzymatic antioxidative systems), determine the stressed state of the affected plants as well as their acclimation or death [18,20,21]. The signaling effect of some ROS like hydrogen peroxide (H_2_O_2_), glutathione and phytohormones as well as the cross-talk between them play an essential role in stress sensing, and induction of different response mechanisms in addition to plant development [18,22,23,24]. Some phytohormones were already tested and proven to alleviate the negative effects due to glyphosate treatment. It was reported that 4PU-30 (phenylurea cytokinin) and salicylic acid applied in maize and barley, respectively partially protected plants from the effects of consecutive glyphosate treatment [14,17]. Recently synthetic auxins were shown to possess high potential for sustaining plant fitness in glyphosate-treated pea [25]. Gibberellic acid application in glyphosate resistant cotton was described to reverse partially the reduction of pollen viability caused by the herbicide [26]. Brassinosteroids are versatile phytohormones which control plant cell growth, physiology and development, regulate reaction to stress including xenobiotic application and are claimed to be eco-friendly compounds [27,28,29]. They are polyhydrohylated steroid hormones (5-cholestane skeleton) divided in three main subclasses depending on the carbon number of the molecule (C27, C28 and C29) [30]. Brassinosteroids are known to be able to alleviate noxious effects of different xenobiotics due to their ability to activate antioxidant activities and to increase pesticide metabolism and detoxification [28,31,32,33,34]. The compound 24-epibrassinolide (EBL) is one of the four major brassinosteroids found in plants, which is often used in physiological research [28,29,33].

To our knowledge, there is no documented study investigating the effects of exogenous EBL pretreatment on wheat plants subjected to subsequent foliar glyphosate application. The present study aims to expand the knowledge of the potential of EBL pretreatment to mitigate negative effects from foliar glyphosate application on wheat through assessment of growth parameters, stress markers (malondialdehyde, MDA; H_2_O_2_ and proline) and non-enzymatic and enzymatic antioxidant system.

## 2. Materials and Methods

### 2.1. Experimental Model

Winter wheat (*Triticum aestivum* L., cv. Sadovo-1) seeds were purchased from the Institute of Plant Genetic Resources “Konstantin Malkov” (Sadovo, Bulgaria). The cultivar is characterized by high ecological plasticity and stable yield and is widely used in the national agriculture sector. Wheat seedlings were grown under controlled conditions (22/17 °C day/night temperatures, 16/8 h day/night photoperiod, 60% relative air humidity) as a hydroponic culture.

Wheat leaves were sprayed with a 1 µM 24-epibrassinolide (EBL) at 13th day after germination. Twenty four hours later seedlings were sprayed with 0.5 mM glyphosate solution (dissolved in distilled water supplied with 0.1% (*v*/*v*) Tween 80 as surfactant). Foliar treatments were performed by a sprayer (using 30 mL solution) until the shoots become completely wet. The control plants were sprayed with distilled water containing 0.1% (*v*/*v*) Tween 80. Growth (length and fresh weight) of roots and shoots as well as some biochemical traits as stress markers (H_2_O_2_, MDA, free proline) non-enzymatic (free-thiol groups-containing and total phenolic compounds) and enzymatic (superoxide dismutase, SOD; catalase, CAT; guaiacol-peroxidase, POX; glutathione-reductase, GR; glutathione S-transferase, GST) activities were examined 14 days after the herbicide application.

### 2.2. Measurements of Growth Parameters

Fresh weight (FW) and shoot and root length were measured fourteen days after glyphosate application. The parameters were evaluated immediately after biomass collection. Length of root and shoot was measured by a linear ruler. The fresh biomass was weighted by electronic balance (Precision Standard TS4000, OHAUS^®^, Parsippany, NJ, USA). The root weight was measured after gentle absorption of the liquid by filter paper.

### 2.3. Biochemical Analyses

Leaf samples were collected from aboveground part of three plants, folded in aluminum foil and stored in liquid nitrogen until analyses. All extractions and biochemical analyses were done at 4 °C. Approximately 250 mg of leaf material was grinded in 4 mL of 0.1% ice cold trichloroacetic acid, centrifuged at 15,000× *g* (4 °C) and the supernatant was used for stress marker and non-enzymatic antioxidant analyses.

#### 2.3.1. Quantification of Stress Markers (H_2_O_2_, MDA, Free Proline)

The amount of H_2_O_2_ was analyzed according to Alexieva et al. [35] and calculated by a standard curve. The reaction mixture of 75 µL supernatant and 75 µL 1M KI was incubated in darkness for 1 h and the absorbance was read at 390 nm. MDA content was measured according to Kramer et al. [36]. The reaction mixture absorbance was read at 532 nm and 600 nm after 45 min incubation with thiobarbituric acid at 100 °C. The amount of MDA was calculated using an extinction coefficient (155 mM^−1^ cm^−1^). Free proline was determined after derivatization with acid ninhydrin reagent for 1 h at 100 °C. Proline content was calculated by a standard curve [37].

#### 2.3.2. Quantification of Non-Enzymatic (Free-Thiol Groups-Containing and Total Phenolic Compounds) Antioxidants

The quantity of free thiols was determined by incubation of 40 µL of supernatant with 150 µL Ellman’s reagent for 10 min at room temperature. The optical density of the reaction mixture was read at 412 nm [38]. The total phenolics were measured by the Folin-Ciocalteu reagent using the method described by Swain and Goldstein [39]. The amount of total phenolics was expressed as gallic acid equivalents.

#### 2.3.3. Quantification of Enzymatic Activities (SOD, CAT, POX, GR, GST)

Approximately 200 mg of shoots were grinded in 3 mL of 100 mM potassium phosphate buffer (4 °C, pH 7.0) containing ethylenediaminetetraacetic acid (1 mM) and polyvinylpyrrolidone (1%).

Superoxide dismutase activity was measured by following the photochemical reduction of nitroblue tetrazolium at 560 nm. One unit of SOD is the amount of enzyme necessary to cause 50% inhibition of the reaction [40]. Catalase activity was determined according to Aebi [41]. The rate of degradation of H_2_O_2_ was monitored at 240 nm. Peroxidase activity was measured by using guaiacol as an electron donor according to Dias and Costa [42]. The change in the optical density was followed at 470 nm. The activity of glutathione reductase was measured by the procedure described by Smith et al. [43]. The rate of reduction of oxidized gluthatione was monitored at 412 nm. Gluthatione S-transferase activity was determined by measuring the rate of conjugation of GSH with 1-chloro-2,4-dinitrobenzene. The reaction was monitored at 340 nm [44].

### 2.4. Statistical Analysis

The experiments were repeated three times. Each sample was collected in three replicates for biochemical analyses and in ten replicates for growth traits. One-way ANOVA with post-hoc Duncan’s multiple range test (*p* < 0.05) was used to assess the significant differences between treatment groups. The figures present mean values ± SE.

## 3. Results

### 3.1. Effect on Wheat Growth

**Figure 1:**
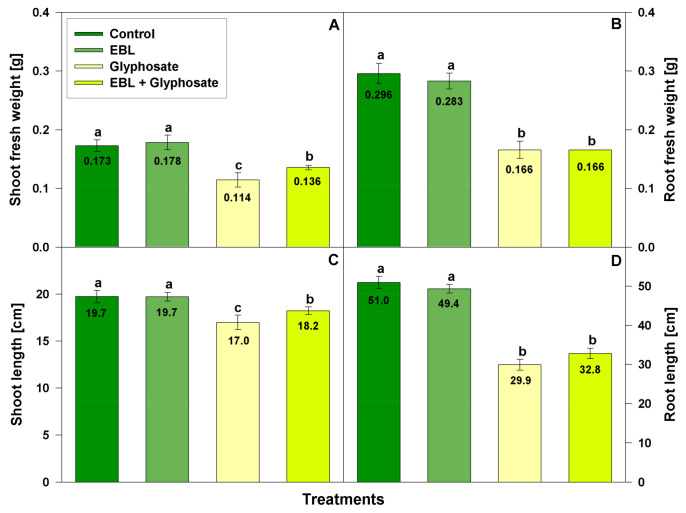
Fresh weight of shoot (**A**) and root (**B**), and length of shoot (**C**) and root (**D**) of 28-day-old wheat plants after foliar pretreatment with 1 µM EBL and subsequent exposure to 0.5 mM glyphosate. EBL stands for 24-epibrassinolide. The numbers within bars represent mean values. The different small letters designate statistical significance among the treatment groups (*n* = 30, *p* < 0.05).

The effects on fresh weight and length of shoots and roots are presented on Figure 1. EBL application did not affect shoot and root growth of wheat plants which remained similar to the control. The negative effect of glyphosate on wheat growth was stronger in roots (46% inhibition of FW and 41% of the organ’s length) than in shoots (34% reduction of FW and 14% length inhibition). When EBL was applied before glyphosate it significantly mitigated the inhibitory effect of herbicide mainly in the shoots (22% FW and 8% length reduction). EBL pretreatment did not influence the reduction of root growth as a consequence of the herbicide treatment, and it remained 45% and 36% inhibition for the FW and the length respectively.

### 3.2. Content of Stress Markers

**Figure 2:**
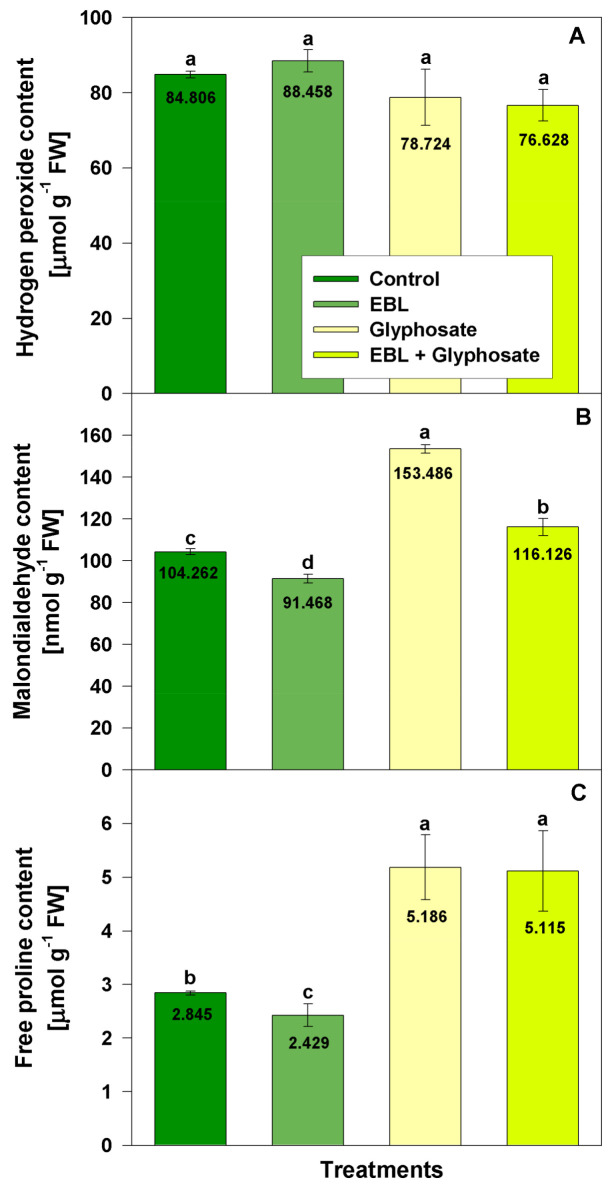
Content of hydrogen peroxide (**A**), malondialdehyde (**B**), and free proline (**C**) measured in the leaves of 28-day-old wheat plants after foliar application of 1 µM EBL and subsequent exposure to 0.5 mM glyphosate. EBL stands for 24-epibrassinolide. The numbers within bars represent mean values. The different small letters designate statistical significance among the treatments (*n* = 9, *p* < 0.05).

In order to evaluate the effect of EBL and glyphosate, we monitored the amounts of hydrogen peroxide, malondyaldehyde (MDA), and proline in the shoots (Figure 2). The applied treatments did not significantly alter H_2_O_2_ levels.

EBL and glyphosate had opposite effect on MDA content. The brassionsteroid treatment caused a slight reduction (by 12%) while the herbicide led to a significant increase with 47% as compared to control. In the EBL-pretreated plants, the measured MDA amount after the application with glyphosate dropped but still was slightly incremented by 11% as compared to control.

Proline level was moderately reduced (15%) by EBL application and significantly increased by the glyphosate treatment (82%). The pretreatment with EBL did not affect the high proline levels resulting from the subsequent treatment with glyphosate (80%).

### 3.3. Content of Non-Enzymatic Antioxidants

**Figure 3:**
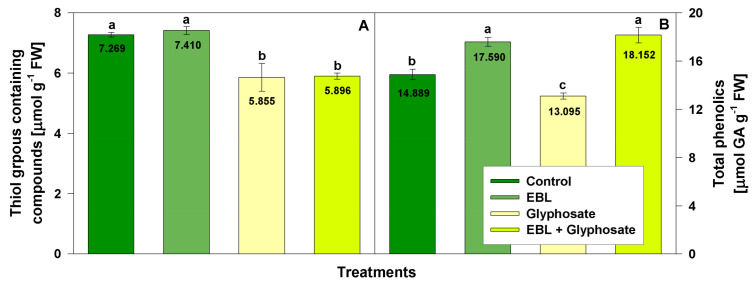
Content of thiol groups containing compounds (**A**) and total phenolic compounds (**B**) measured in the leaves of 28-day-old wheat plants after foliar application of 1 µM EBL and subsequent exposure to 0.5 mM glyphosate. EBL stands for 24-epibrassinolide, GA—gallic acid. The numbers within bars represent mean values. The different small letters indicate statistical significance among the treatment groups (*n* = 9, *p* < 0.05).

The levels of low molecular thiol-groups containing compounds and total phenolics were examined to assess the effect of EBL and herbicide on non-enzymatic antioxidant defense system (Figure 3).

Compared to the control levels, EBL did not alter the content of thiol-groups containing compounds. As expected, an inhibition of thiols (by 19%) was observed after glyphosate and EBL + glyphosate treatments.

Phenolics were slightly induced (by 18%) by EBL treatment and their levels were negatively influenced by the glyphosate application (12%). The combined treatment significantly increased the amount of total phenolics as compared to the control (22% above the control).

### 3.4. Effect on Enzymatic Antioxidant Activities

**Figure 4:**
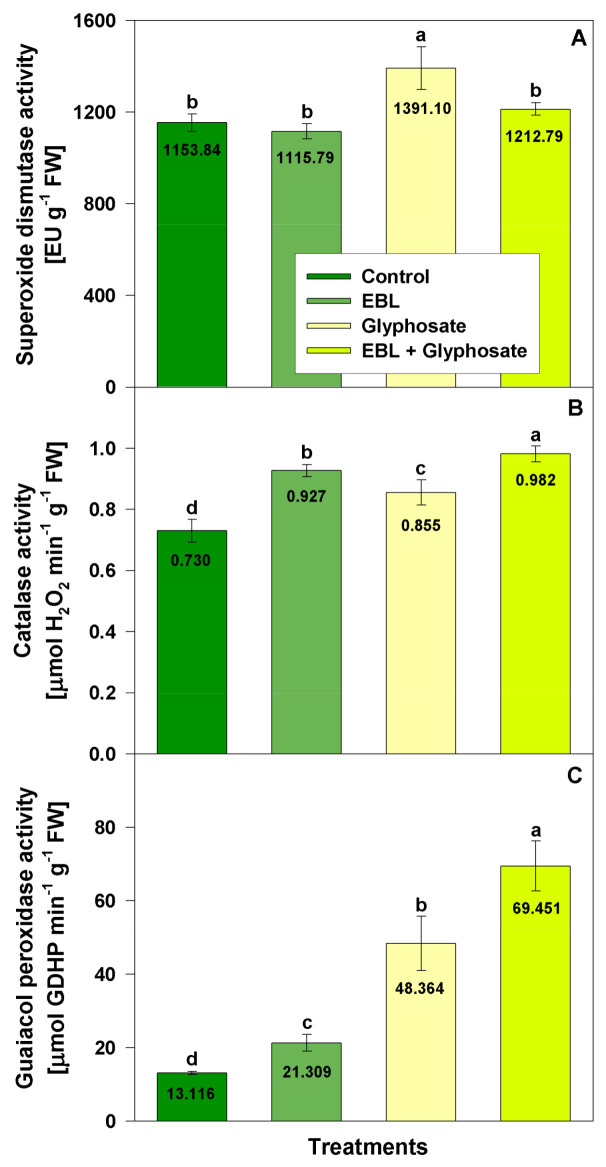
Activity of superoxide dismutase (**A**), catalase (**B**) and guaiacol peroxidase (**C**) in the leaves of 28-day-old wheat plants after foliar application of 1 µM EBL and subsequent exposure to 0.5 mM glyphosate. EBL stands for 24-epibrassinolide. The numbers within bars represent mean values. The different small letters indicate statistical significance among the treatments (*n* = 9, *p* < 0.05).

**Figure 5:**
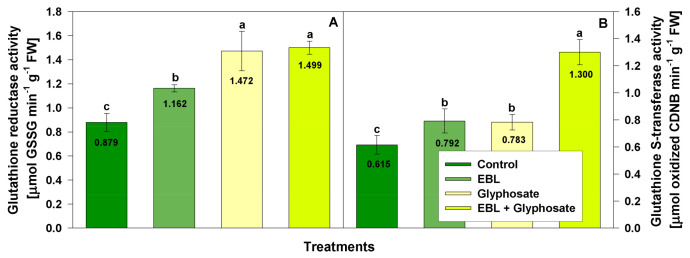
Activity of glutathione reductase (**A**) and glutathione S-transferase (**B**) in the shoots of 28-day-old wheat plants after foliar application of 1 µM EBL and subsequent exposure to 0.5 mM glyphosate. EBL stands for 24-epibrassinolide, GSSG—oxidized glutathione. The numbers within bars represent mean values. The different small letters indicate statistical significance among the treatments (*n* = 9, *p* < 0.05).

To assess the effects of EBL and glyphosate on the enzymatic antioxidant defense system several essential enzymatic activities as catalase, guaiacol peroxidase, superoxide dismutase (Figure 4), glutathione S-transferase and glutathione reductase (Figure 5) were measured.

Superoxide dismutase (SOD) activity was not altered by EBL applied alone or in combination with glyphosate (Figure 4A). Only glyphosate treatment caused slight induction of SOD activity (21%).

The EBL application slightly increased CAT activity (Figure 4B) when applied alone (27%) or in combination with glyphosate (35%). A raise in CAT activity after glyphosate treatment was observed as well (17%).

Peroxidase activity was the most significantly affected enzyme in the current experimental model. EBL pretreatment enhanced its activity by 62%, glyphosate application by 269% and EBL + glyphosate by 430% (Figure 4C). As anticipated, an induction of GR and GST activities was found after glyphosate and EBL applications (Figure 5). GR activity increased slightly after EBL (32%) and more substantially after glyphosate (67%) and EBL + glyphosate (71%) application (Figure 5A). The activity of GST was enhanced slightly by EBL (29%) and by glyphosate (27%) application but the combined (EBL + glyphosate) treatment doubled the enzymatic activity as compared to the control (Figure 5B).

## 4. Discussion

The shikimate pathway is known to be the main target in glyphosate-treated plants. Oxidative events and/or oxidative stress were documented as secondary effects resulting from herbicide application, but presently the generation of oxidative damage is well acknowledged as a major detrimental consequence due to glyphosate [5,13,14,15,16,17,45]. Hydrogen peroxide, superoxide anion and hydroxyl radical are the ROS reported to have negative impact after glyphosate application [5,10,13,17]. Their overproduction is known to cause oxidative injury to macromolecules and biomembranes [32,34,46,47]. Besides EBL [28,32,47], pretreatment with BR mimetics (bikinin and brazide) also enhanced growth traits, photosynthesis, antioxidant enzyme activities, expression of glutathione-related genes and reduced substantially ROS levels and herbicide residues in plants [16]. EBL is anticipated to take part in both induction of herbicide detoxification and activation of antioxidant defense systems [28,32,48].

The focus in current investigation is on the effects on some universal stress markers (MDA, H_2_O_2_, proline) indicating predominantly plant oxidative status, and on the antioxidative defense system (non-enzymatic and enzymatic). Low-molecular thiols and total phenolics were explored as commonly induced members of the non-enzymatic antioxidant defense while SOD, CAT, GR, and POX were monitored as the major participants in the enzymatic defense systems along with GST, which is involved in xenobiotic detoxification.

The negative effect of glyphosate on growth is expected as the inhibition of the shikimate pathway affects not only the biosynthesis of aromatic amino acids, but also a number of secondary metabolites of phenolic nature, including auxins [49]. The inhibition of plant growth as a result of glyphosate application has been well-documented [5,12,14,16,17,50]. Glyphosate or its metabolites are known to translocate to organs and tissues with intense metabolism like the meristems in the root and the shoot tips. This could be linked to the observed strong inhibitory effect on the root growth after the foliar application of the herbicide [10,12].

EBL-priming of wheat plants (24 h before herbicide application) appeared to be able to mitigate partially the negative consequences of the oxidative stress caused by the subsequent glyphosate treatment. This was manifested by improved shoot growth of the plants after the combined treatment. The effect on oxidative stress markers (proline, MDA and H_2_O_2_) requires a critical interpretation considering various factors (stress intensity and duration, plant species and growth phase etc.) that may alter their levels in different manner depending on the developmental and environmental circumstances. For example, MDA as the final product of lipid peroxidation has been reported to increase due to glyphosate application in wheat, pea, maize, *Salvinia natans* (L.), *Hordeum vulgare* L, *Vallisneria natans* (Lour.) Hara, and tomato roots [5,11,14,16,17,45,51]. An opposite trend has been observed in other studies in which the excessive herbicide concentrations caused a reduction and a tissue-specific decrease of MDA [5]. Both inhibition and induction of H_2_O_2_ content were also observed after glyphosate application [14,16,17,45,52]. In willow leaves and *S. lycopersicum* roots lack of alteration in hydrogen peroxide level due to glyphosate treatment was also documented [5,11]. In addition to its role in the primary protein structure, proline is important compatible solute, able to scavenge ROS, but often it is discussed also as a stress indicator [53]. Generally, the proline amount increases in glyphosate-treated plants [5,14,17,54] and the results presented here are in line with these observations. Hydrogen peroxide and proline have a double function in stress response. As a secondary messenger, the mild increase of H_2_O_2_ may activate different sets of transcription factors, the antioxidant defense system and/or phytohormones. Hydrogen peroxide is regarded as the most potent ROS which is capable to normalize the redox homeostasis state [46,55]. The absence of H_2_O_2_ induction in glyphosate-treated wheat plants could be due to the activation of the antioxidative enzymes, with the considerable increase of peroxidase activity in particular, and to a possible boost of superoxide anion which has been observed in other model systems [5,17]. The raise in SOD activity after herbicide treatment might be linked to the augmentation of superoxide radical.

The significant increment of MDA indicates a strong imbalance of the biomembrane lipid peroxidation in the herbicide-treated plants. Here we demonstrate that the pretreatment with EBL prior glyphosate application mitigates the negative effect of the herbicide on lipid peroxidation as MDA content almost drops down to the control level. The reduction of the lipid peroxidation in the experimental group subjected to the consecutive application of EBL and glyphosate, judging from the close to the control MDA levels, suggests positive effect of the administered pretreatment. The reduced levels of the monitored stress markers after EBL application is not surprising as brassinosteroids are known to affect positively plant’s metabolism during acclimation to stress [56,57]. The reduction of oxidative stress caused by various pesticides due to EBL application was observed in different model systems [28,34,47].

The decrease in thiols after glyphosate application might be considered as another symptom of oxidative stress resulting from the disturbed redox homeostasis [24]. Glutathione is determined as the main compound within low-molecular-weight thiols [46,58]. The reduction in thiols in parallel with the raise in GST activity might be associated with phase II of xenobiotic detoxification [59]. The decline in the content of phenolic compounds most probably is due to Shikimate pathway inhibition which is the main glyphosate target. The enhancement of phenolics found after EBL and EBL + glyphosate indicate an activation of the non-enzymatic defense system as these compounds are recognized as ROS scavengers [60].

The increase in the activities of antioxidant (POX, CAT, GR) and detoxification (GST) enzymes found in EBL- or glyphosate-treated plants were previously reported [14,32,45,47,61]. The present research demonstrates that POX was the most affected antioxidant enzymatic activity by glyphosate treatment and this effect was strengthened after the EBL + glyphosate application. Similar alterations in the content of H_2_O_2_, and the activities of POX and CAT were reported earlier in *Brassica juncea* L. or rice plantlets [34,47] after EBL seed-priming. The increased activity of POX could be an important element of xenobiotics metabolism [32,61]. In general, peroxidases are able to take part in both H_2_O_2_ augmentation or utilization trough oxidative cycle or peroxidative cycle, respectively [62,63]. In the classical peroxidative cycle H_2_O_2_ is used to transform phenolic substrate compounds into corresponding phenoxy radicals which can consequently make final products as lignins [62,63]. In the oxidative cycle NADH is oxidized as electron donor to NAD^•^ radical, which subsequently reduces the oxygen to superoxide radical [63]. Subsequently the ‘‘NADH-oxidase’’ activity of peroxidase results in H_2_O_2_ synthesis with or without the contribution of SOD [62]. However, the slight induction of SOD after herbicide treatment in our study indicates the necessity the superoxide radical to be scavenged in these plants.

EBL pretreated plants were previously reported to increase *GST* expression or GST activity in combination with other pesticides or xenobiotics [32,34]. The presented results show that GST was involved in the detoxification process in the EBL pretreated experimental group. The stunted growth and increased lipid peroxidation caused by glyphosate was partially alleviated by the EBL pretreatment. This was accompanied by increased total phenolics content and higher activities of POX, CAT, GST and GR.

## 5. Conclusions

We confirmed that glyphosate treatment reduces substantially wheat growth and inhibits the non−enzymatic defense (thiols and phenolic compounds) of the affected plants. The herbicide negative influence on the physiological status of the plants is manifested by the increased amounts of the stress markers proline and MDA and the activation of the antioxidant and the xenobiotic detoxification enzymes. Applied alone EBL slightly reduced stress markers (MDA and proline), enhanced phenolic compounds and induced enzymatic defense system (POX, GR, GST and CAT). We demonstrate that EBL pretreatment improves the performance of wheat plants subjected to glyphosate as evidenced by the partially restored shoot growth and the reduction of the lipid peroxidation marker MDA. Our data support earlier findings that brassinosteroids promote the metabolism of pesticides and are crucial for their proper detoxification [32,33,61]. It could be concluded that the exogenous EBL induces xenobiotic detoxification through activation of POX and GST. As a result the activation of the antioxidant defense probably exerts a protective role towards the negative effects of the glyphosate action.

## Data Availability

Not applicable.
